# Prognostic significance of IL‐18 in acute coronary syndrome patients

**DOI:** 10.1002/clc.24229

**Published:** 2024-01-29

**Authors:** Chenchun Xiong, Qiaoting Yu, Feng Gao, Song Liu, Jianhui Zhang, Tianyi Ma, Suifeng Liu

**Affiliations:** ^1^ Department of Cardiology, School of Medicine, Zhongshan Hospital of Xiamen University Xiamen University Xiamen Fujian China; ^2^ Shengli Clinical Medical College Fujian Medical University Fuzhou Fujian China; ^3^ Hospital of Guizhou Medical University Guiyang Guizhou China

**Keywords:** acute coronary syndrome, adverse clinical events, inflammation, interleukin‐18, interleukin‐1β, prognosis

## Abstract

**Background:**

After acute coronary syndrome (ACS), inflammation aids healing but may harm the heart. Interleukin (IL)‐18 and IL‐1β are pivotal proinflammatory cytokines released during pyroptosis, a process that initiates and sustains inflammation. This study aimed to evaluate the levels of circulating IL‐18 and IL‐1β during the progression of ACS and to determine their association with subsequent clinical events in ACS patients.

**Hypothesis:**

Circulating levels of IL‐18 and IL‐1β are associated with subsequent clinical events in ACS patients.

**Methods:**

Employing immunoassays, we examined plasma levels of IL‐1β and IL‐18 in 159 ACS patients and matched them with 159 healthy controls. The primary composite endpoint included recurrent unstable angina, myocardial infarction, heart failure exacerbation, stroke, or cardiovascular death.

**Results:**

ACS patients exhibited a significant increase in plasma IL‐18 levels, measuring 6.36 [4.46−9.88] × 10^2^ pg/mL, in contrast to the control group with levels at 4.04 [3.21−4.94] × 10^2^ pg/mL (*p* < 0.001). Conversely, plasma levels of IL‐1β remained unchanged compared to the control group. Following a 25‐month follow‐up, IL‐18 levels exceeding the median remained an important prognostic factor for adverse clinical events in ACS patients (hazard ratio = 2.37, 95% confidence interval: 1.14−4.91, *p* = 0.021). Besides, IL‐18 displayed a nonlinear association with adverse clinical events (*p* nonlinear = 0.044). Subgroup analysis revealed that the correlation between IL‐18 and the risk of adverse clinical events was not significantly affected by factors such as age, sex, history of diabetes, smoking, Gensini score, or ACS type (all *p* interaction >0.05).

**Conclusion:**

IL‐18 appears to hold potential as a predictive marker for anticipating clinical outcomes in patients with ACS.

## Graphical abstract

Our findings indicate significantly higher interleukin (IL)‐18 levels in acute coronary syndrome (ACS) patients versus the control group, while IL‐1β levels remained consistent. Notably, we've unveiled a substantial, nonlinear link between elevated IL‐18 levels and increased risk of adverse clinical events in ACS. Subgroup analysis underscored the robustness of these associations.

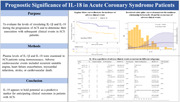

## 1 INTRODUCTION

Acute coronary syndrome (ACS) is a serious condition that often arises when the delicate balance between the instability and healing of atherosclerotic plaques within blood vessels is disrupted.^1^ It imposes a significant global health burden and leads to elevated mortality rates.^2^ Considerable evidence underscores the central role of inflammation in atherosclerosis development, potentially causing plaque destabilization and rupture.^3^, ^4^, ^5^, ^6^ Pyroptosis, a specific form of programmed cell death, is intricately intertwined with inflammatory responses and substantially contributes to the onset and advancement of atherosclerosis through the triggering of multiple signaling pathways. It involves toll‐like receptor stimulation, NOD‐like receptor pyrin domain‐containing protein 3 (NLRP3) complex formation, and caspase 1 activation, leading to interleukin (IL)‐18 and IL‐1β secretion.^7^, ^8^

IL‐18 and IL‐1β play pivotal roles in pyroptosis, intensifying the immune response and inflammation. IL‐1β contributes locally to vascular inflammation and destabilization of atherosclerotic plaques, targeted by drugs like canakinumab in ACS.^9^, ^10^ IL‐18, akin to IL‐1β, sparks inflammation in atherosclerosis and plaque instability.^11^, ^12^ While IL‐1β is a target for anti‐inflammatory therapies, IL‐18's role is intricate. Reports on circulating IL‐18's predictiveness for cardiovascular mortality and endpoints in coronary disease and ACS clash. Baseline IL‐18 levels associated with cardiovascular mortality in PLATelet Inhibition and Patient Outcomes (PLATO) trial, but this weakened after adjusting for other biomarkers.^13^ Serum IL‐18 levels may signal high mortality risk in stable and unstable angina (UA).^14^ Elevated IL‐18 in ACS links to long‐term all‐cause and non‐cardiovascular mortality, warranting further comprehensive research into its ACS implications.^15^

The present study involved a cohort of ACS patients whose medical progress was tracked over a median duration of 25 months. The objective was to explore the correlations between IL‐1β and IL‐18 levels and the likelihood of experiencing mortality and cardiovascular‐related morbidity over an extended follow‐up period.

## 2 METHODS

### 2.1 Study design and population

This observational study enrolled 197 patients diagnosed with ACS who were admitted to Zhongshan Hospital of Xiamen University from May 2020 to March 2021. The patients were diagnosed with UA, non‐ST‐segment elevation myocardial infarction (NSTEMI), or ST‐segment elevation myocardial infarction (STEMI) based on the 2023 ESC Guidelines on Acute Coronary Syndrome.^16^ Exclusion criteria were applied, leading to the exclusion of 38 patients due to various conditions, including severe infections, tumors, hematological disorders, immune disorders, severe liver diseases, chronic kidney failure requiring dialysis, glomerular filtration rate <30 mL/min, congenital heart defects, mental illness, or noncooperation. A healthy control group, consisting of 159 age‐ and sex‐matched individuals who underwent a physical examination during the same period, was also included in the study. Ethical approval was obtained from the Ethics Committee of Zhongshan Hospital affiliated with Xiamen University (approval number: xmzsyyky, 2021‐141).

### 2.2 Data collection and laboratory analysis

Blood samples were meticulously collected from all participants on the initial postoperative day after undergoing coronary angiography following an overnight fasting period, with strict adherence to standardized conditions. Plasma samples were immediately centrifuged at 3000 rpm for 10 min at 4°C, divided into aliquots, and stored at −80°C until analysis. Various laboratory parameters, including white blood cells (WBCs), neutrophils (NEUTs), C‐reactive protein (CRP), d‐dimer, N‐terminal pro‐B‐type natriuretic peptide (NT‐proBNP), high‐sensitivity cardiac troponin T (hsCTnT), fibrinogen, international normalized ratio (INR), aspartate aminotransferase (AST), alanine aminotransferase (ALT), creatinine (Cr), hemoglobin A1c (HbA1c), and lipid serum levels were measured using standard methodologies. Plasma IL‐18 and IL‐1β levels were measured via enzyme‐linked immunosorbent assays obtained from R&D Systems.

### 2.3 Cardiovascular evaluation and treatment strategies

Following admission, all patients underwent echocardiography (Vivid E9; GE Healthcare Medical Systems), which involved measuring parameters such as interventricular septum thickness, left atrial diameter (LA), left ventricular end‐systole dimension (LVDs), left ventricular end‐diastolic dimension (LVDd), and left ventricular posterior wall thickness (LVPW). The left ventricular ejection fraction (LVEF) was calculated using biplane Simpson's method. All ACS patients underwent coronary angiography, and the severity of atherosclerosis was assessed using the Gensini score.^17^

Upon discharge, the study population received a standardized treatment protocol for secondary coronary heart disease prevention. However, the prescription of specific medications—such as antiplatelets, beta‐blockers, statins, renin–angiotensin system inhibitors, calcium channel blockers, and anticoagulants—might vary depending on individual patient characteristics and comorbidities.

### 2.4 Statistical analysis

The statistical analysis was performed using R software, version 4.2.2. Descriptive statistics were reported as mean ± standard deviation (x̅±S), medians (with interquartile range), or as percentages (%), as appropriate for each variable. Continuous variables were compared using the Mann‒Whitney *U* test or *t*‐test, while categorical variables were analyzed utilizing the *χ* ^2^ test or Fisher exact test.

ACS participants were stratified into low IL‐18 and high IL‐18 groups based on the median level of IL‐18. Baseline characteristics were compared between these two groups, as well as between participants who experienced adverse clinical events and those who did not. Survival was estimated using the Kaplan‒Meier method, and potential disparities in survival were assessed through a stratified log‐rank test. Cox regression models were used to quantify the association between IL‐18 and adverse clinical events, with results presented as hazard ratios (HRs) and 95% confidence intervals (CI). Two sets of models (Model I and Model II) were used, adjusting for specific variables related to age, gender, diabetes, hypertension, LVEF, NT‐proBNP, hsCTnT, Gensini score, CRP, and d‐dimer. In ensuring the utmost clinical significance within this composite analysis, the win ratio (WR) method was employed to validate the strength and reliability of the findings.^18^ IL‐18 levels were categorized as both continuous and categorical variables, with the median of IL‐18 in each subgroup used as the reference category.

To evaluate dose–response relationships and potential nonlinear associations, restricted cubic splines (RCS) with four knots were employed, with analysis of variance testing for nonlinearity. Upon identifying nonlinearity, segmented regression was employed to establish a piecewise linear relationship. A recursive algorithm was applied to ascertain the inflection point threshold.^19^ Interaction and subgroup analyses were carried out to assess heterogeneity in the effect of IL‐18 on adverse clinical events across different subgroups categorized by age, sex, history of diabetes, hypertension, smoking, Gensini score, and ACS type. A two‐tailed test with a significance level of *p* < 0.05 was employed to ascertain statistical significance in all analyses.

## 3 RESULTS

### 3.1 Baseline characteristics of the study population

The baseline characteristics of the study cohort are detailed in Table 1. Among the entire study cohort, IL‐18 measurements were available for 318 patients, while IL‐1β measurements were limited to 127 patients. Notably, 191 participants had IL‐1β levels under the detection threshold of the assay. Significant differences were noted between the control and ACS groups, particularly in terms of the prevalence of diabetes in their medical histories. Cardiac ultrasound results indicated statistically significant differences in LVPW and LVEF between healthy individuals and ACS patients. Additionally, ACS patients displayed significantly lower levels of HDL cholesterol and elevated levels of apoA1, apoB, IL‐18, CRP, WBC, NEUT, fibrinogen, hsCTnT, NT‐proBNP, INR, d‐dimer, HbA1c, Cr, ALT, and AST compared to the control group (*p* < 0.05).

**Table 1:** Baseline characteristics of the entire study population.

	Control (*n* = 159)	ACS (*n* = 159)	*p*
Age, years	62.98 (12.43)	62.98 (12.43)	1.000
Men, *n* (%)	119 (74.8)	122 (76.7)	0.695
Diabetes, *n* (%)	19 (11.9)	60 (37.7)	<0.001^a^
Hypertension, *n* (%)	77 (48.4)	96 (60.4)	0.635
Stroke, *n* (%)	5 (3.1)	11 (6.9)	0.124
kidney disease, *n* (%)	8 (5.0)	11 (6.9)	0.637
Smoking habit, *n* (%)	56 (35.2)	71 (44.7)	0.086
Drinking habit, *n* (%)	26 (16.4)	35 (22.0)	0.200
Proteinuria, *n* (%)	19 (11.9)	31 (19.5)	0.065
LA, mm	38.00 [35.00−41.25]	39.50 [35.25−42.00]	0.310
IVS, mm	10.25 [9.60−11.80]	10.75 [9.60−12.78]	0.159
LVPW, mm	9.72 ± 1.17	10.03 ± 1.59	0.004^a^
LVDd, mm	48.21 ± 6.25	49.31 ± 5.71	0.985
LVDs, mm	30.78 + 6.51	32.37 ± 5.47	0.376
LVEF, %	66 [60−72]	61 [52−66]	<0.001^a^
Total cholesterol, mmol/L	4.76 [3.92−5.48]	4.93 [3.96−5.82]	0.108
Triglycerides, mmol/L	1.54 [1.04−2.38]	1.51 [1.08−2.42]	0.627
HDL‐c, mmol/L	1.20 ± 0.31	1.09 ± 0.26	0.017^a^
LDL‐c, mmol/L	3.04 ± 0.95	3.32 ± 1.05	0.299
apoA1, g/L	0.93 [0.73−1.09]	0.96 [0.76−1.17]	<0.001^a^
apoB, g/L	0.87 [0.73−1.06]	0.96 [0.80−1.15]	0.016^a^
IL‐18, 10^2^pg/mL	4.04 [3.21−4.94]	6.36 [4.46−9.88]	<0.001^a^
IL‐1β, pg/mL^b^	0.12 [0.06−0.30]	0.15 [0.05−0.48]	0.726
CRP, mg/L	1.70 [0.78−3.71]	4.08 [1.94−18.22]	0.001^a^
WBC, 10^9^/L	6.67 [5.23−7.72]	8.67 [6.98−11.35]	<0.001^a^
NEUT, 10^9^/L	3.90 [3.26−5.02]	6.18 [4.46−9.04]	<0.001^a^
hsCTnT, ng/L	24 [7.80−597.00]	490.10 [101.45−2680.00]	<0.001^a^
NT‐proBNP, ng/L	46.00 [22.25−140.50]	331.00 [78.25−1140.50]	<0.001^a^
Fibrinogen, g/L	3.00 [2.61−3.49]	3.24 [2.76−4.01]	<0.001^a^
INR	0.98 [0.94−1.04]	1.03 [0.97−1.14]	<0.001^a^
d‐dimer, mg/L	0.24 [0.00−0.50]	0.34 [0.20−0.62]	0.009^a^
ALT, U/L	11.40 [27.80−27.28]	25.70 [16.90−43.80]	0.002^a^
AST, U/L	22.35 [18.23−26.85]	39.30 [23.35−150.45]	<0.001^a^
HbA1c, %	5.90 [5.60−6.20]	6.30 [5.80−7.30]	<0.001^a^
Cr, µmol/L	76.25 [63.85−89.98]	79.90 [65.40−94.50]	0.162
Gensini score	n.d.	55.00 [33.00−81.50]	

Abbreviations: ACS, acute coronary syndrome; ALT, alanine transaminase; apo A1, apolipoprotein A1; apo B, apolipoprotein B; AST, aspartate transaminase; Cr, blood creatinine; CRP, C‐reactive protein; HbA1C, glycosylated hemoglobin; HDL‐c, high‐density lipoprotein cholesterol; hsCTnT, high‐sensitive cardiac troponin; IL‐18, interleukin 18; INR, international normalized ratio; IVS, interventricular septum; LA, left atrial diameter; LDL‐c, low‐density lipoprotein cholesterol; LVDd, left ventricular end diastolic dimension; LVDs, left ventricular end systolic diameter; LVEF, left ventricular ejection fraction; LVPW, left ventricular posterior wall; NEUT, neutrophil count; NT‐proBNP, N‐terminal pro‐B‐type natriuretic peptide; WBC, white blood cell.

^a^ Statistically significant.

^b^ Not detectable (0.033 pg/mL) in 191 (108 in the control group and 83 in the ACS group) out of 318 participants; excluded from the analyses.

Unlike IL‐18, plasma levels of IL‐1β didn't exhibit significant changes in comparison to the control group. However, the exclusion of missing IL‐1β values in our analysis raised concerns about potential bias and reduced statistical power. To mitigate this, we conducted a sensitivity analysis, encompassing both best‐ and worst‐case scenarios.^20^ In the best‐case scenario, these values were treated as equivalent to the limit of detection (LOD), while in the worst‐case scenario, they were assumed to be half of the LOD. Following this adjustment, the medians of the revised data sets were compared between groups. Despite these considerations, differences in IL‐1β between the groups did not achieve statistical significance (Supporting Information S1: Table 1).

### 3.2 Comparisons between participants with ACS concerning IL‐18 levels and the occurrence of adverse clinical events

In this cohort study, 41 out of the 159 participants experienced adverse clinical events over a median follow‐up duration of 25 months, including cardiovascular‐related fatalities (4 cases), acute myocardial infarction (AMI) (3 cases), nonfatal strokes (4 cases), heart failure exacerbation (16 cases), and instances of recurrent UA (14 cases). Participants were stratified into two groups according to the median IL‐18 level: the low IL‐18 group (IL‐18 ≤ 6.36 × 10^2^ pg/mL) and the high IL‐18 group (IL‐18 > 6.36 × 10^2^ pg/mL). A comparative analysis of the baseline characteristics of these two groups is presented in Supporting Information S1: Table 2. The findings suggested that the high IL‐18 group exhibited a significantly higher incidence of adverse clinical events and elevated d‐dimer levels compared to the low IL‐18 group. However, no significant disparities were observed in other baseline variables between the two groups.

Supporting Information S1: Table 3 displays a comparison between participants who experienced adverse clinical events and those who did not. Participants who experienced subsequent clinical events had significantly higher IL‐18 levels and Gensini scores, while their LVEF was significantly lower, and they had a thinner LVPW compared to those who did not experience adverse clinical events at baseline.

**Figure 1:**
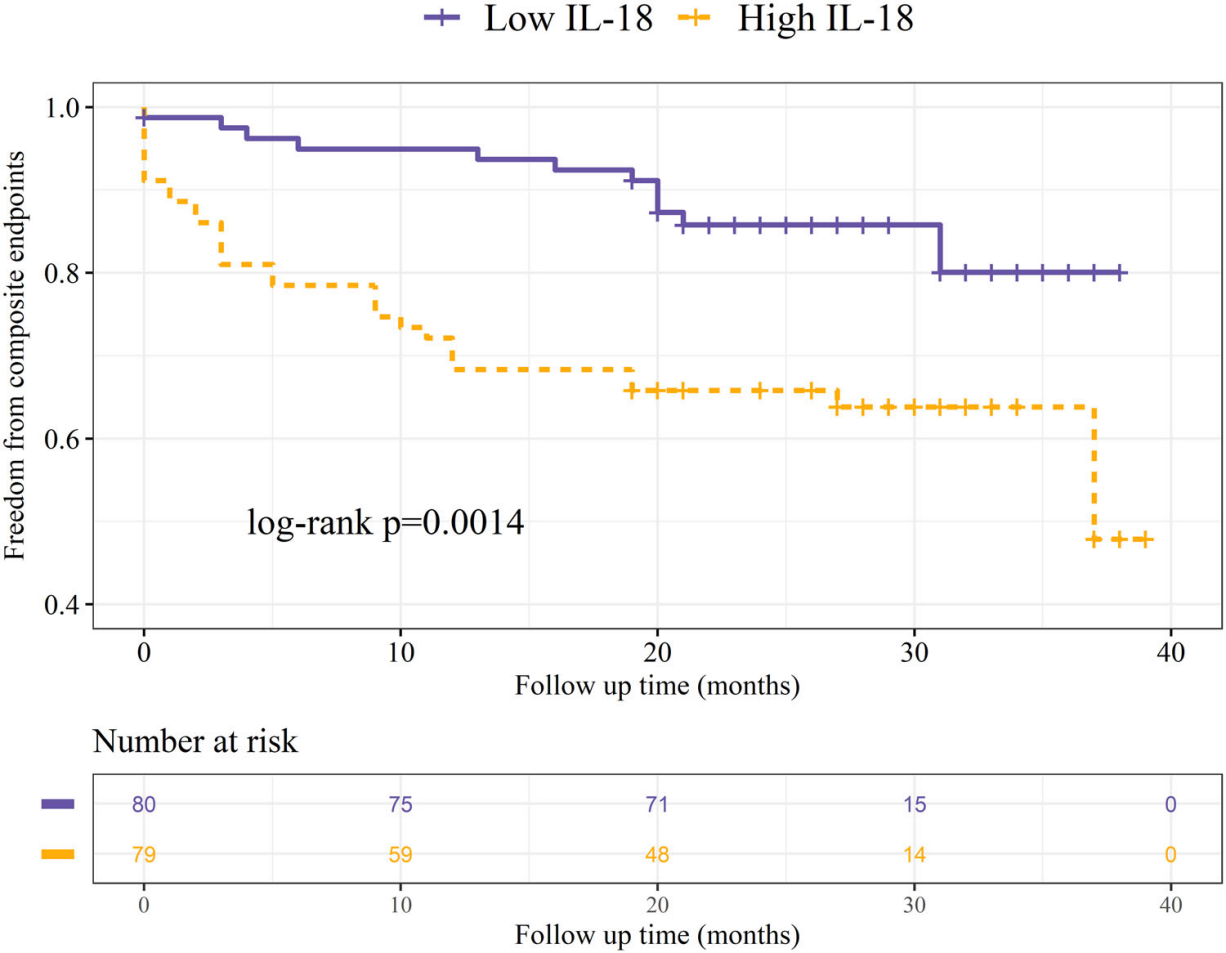
Kaplan−Meier curves depict adverse clinical events in relation to IL‐18 levels within ACS. The curves represent the occurrence of adverse clinical events over a 25‐month follow‐up, categorized by high and low IL‐18 levels. ACS, acute coronary syndrome.

The Kaplan−Meier survival plot demonstrates a noteworthy dissimilarity in the occurrence of adverse clinical events between the high IL‐18 and low IL‐18 groups. This dissimilarity is further confirmed by the Log‐rank test, indicating a highly significant disparity (*p* = 0.0014, Figure 1).

### 3.3 Univariate and multivariate analyses of factors associated with the risk of adverse clinical events

In the univariate Cox regression analysis, it was observed that individuals with higher levels of IL‐18, CRP, NT‐proBNP, d‐dimer, and Gensini scores were at an elevated risk of experiencing adverse clinical events, while higher levels of LVEF were associated with a protective effect against adverse clinical events. In the subsequent multivariate Cox regression analysis, it was reaffirmed that the high IL‐18 group carried a significantly greater risk of adverse clinical events in comparison to the low IL‐18 group (HR = 2.37, 95% CI: 1.14−4.91, *p* = 0.021). Even when IL‐18 was treated as a continuous variable, its association with the risk of subsequent clinical events remained statistically significant (HR = 1.04, 95% CI: 1.00−1.07, *p* = 0.027). Moreover, it was revealed that a higher LVEF level exerted a protective influence on the incidence of adverse clinical events (HR = 0.97, 95% CI: 0.94−1.00, *p* = 0.031). However, no other variables demonstrated a significant impact on adverse clinical events in the multivariate analysis (Table 2).

**Table 2:** Cox regression showing the predictive effects of different variables on adverse clinical events occurrence.

Characteristics	Univariate analysis		Model I		Model II	
	HR (95% CI)	*p*	HR (95% CI)	*p*	HR (95% CI)	*p*
Sociological characteristics						
Age, years	1.02 (0.99−1.05)	0.126	1.02 (0.99−1.05)	0.122	1.02 (0.98−1.05)	0.336
Gender (men)	1.40 (0.62−3.16)	0.422	1.97 (0.83−4.66)	0.125	2.39 (0.90−6.39)	0.082
Smoking	1.00 (0.54−1.85)	0.991	0.93 (0.48−1.80)	0.837	0.76 (0.38−1.52)	0.437
Comorbidities						
Hypertension	1.01 (0.54−1.9)	0.970	1.15 (0.57−2.30)	0.696	1.08 (0.53−2.19)	0.841
Diabetes	1.69 (0.92−3.13)	0.091	1.36 (0.72−2.56)	0.344	1.27 (0.64−2.50)	0.495
kidney disease	1.90 (0.97−3.72)	0.063	1.66 (0.80−3.42)	0.173	1.62 (0.71−3.68)	0.250
Echocardiography						
LA, mm	1.05 (0.99−1.11)	0.076	1.01 (0.95−1.08)	0.681	1.02 (0.95−1.09)	0.533
LVDs, mm	1.04 (0.99−1.09)	0.108	0.99 (0.94−1.05)	0.804	1.00 (0.94−1.06)	0.889
LVEF, %	0.96 (0.94−0.98)	0.001^a^	0.96 (0.94−0.99)	0.003^a^	0.97 (0.94−1.00)	0.031^a^
Laboratory values						
Cardiac function parameters						
hsCTnT, pg/mL	1.00 (1.00−1.00)	0.758	1.00 (1.00−1.00)	0.953	1.00 (0.99−1.01)	0.454
NTproBNP, 10^3^pg/mL	1.06 (1.02−1.10)	0.005^a^	1.05 (0.99−1.11)	0.095	1.04 (0.98−1.10)	0.231
Inflammatory markers						
IL‐18, 10^2^pg/mL	1.03 (1.00−1.06)	0.036^a^	1.03 (1.00−1.07)	0.044^a^	1.04 (1.00−1.07)	0.027^a^
IL‐18 ≤median	Ref.	Ref.	Ref.	Ref.	Ref.	Ref.
IL‐18 >median	2.87 (1.46−5.63)	0.002^a^	2.39 (1.20−4.79)	0.014^a^	2.37 (1.14−4.91)	0.021^a^
WBC, 10^9^/L	1.05 (0.97−1.13)	0.214	1.04 (0.96−1.12)	0.328	1.02 (0.93−1.11)	0.719
CRP, mg/L	1.01 (1.00−1.01)	0.008^a^	1.00 (1.00−1.01)	0.262	1.00 (0.99−1.01)	0.705
Coagulation parameters						
INR	0.71 (0.21−2.4)	0.576	0.63 (0.13−3.00)	0.565	0.54 (0.09−3.41)	0.516
Fibrinogen, g/L	1.05 (0.80−1.37)	0.725	0.92 (0.67−1.26)	0.604	0.71 (0.47−1.06)	0.096
d‐dimer <median	Ref.	Ref.	Ref.	Ref.	Ref.	Ref.
d‐dimer ≥median	2.26 (1.22−4.17)	0.010^a^	1.85 (0.95−3.61)	0.070	1.59 (0.76−3.3)	0.217
Lipid profile						
HDL‐c, mmol/L	0.75 (0.22−2.60)	0.652	0.72 (0.19−2.69)	0.628	0.71 (0.19−2.72)	0.619
LDL‐c, mmol/L	0.92 (0.68−1.25)	0.603	0.98 (0.71−1.36)	0.922	1.09 (0.76−1.57)	0.646
apoA1, g/L	0.31 (0.08−1.18)	0.087	0.48 (0.11−2.03)	0.320	0.71 (0.15−3.33)	0.661
Other parameters						
Cr, µmol/L	1.00 (1.00−1.01)	0.270	1 (0.99−1.01)	0.626	0.99 (0.98−1.01)	0.388
HbA1C, %	1.06 (0.91−1.22)	0.473	0.99 (0.81−1.2)	0.884	1.00 (0.81−1.24)	0.980
Gensini	1.01 (1.00−1.02)	0.041^a^	1.01 (1.00−1.01)	0.236	1.00 (0.99−1.01)	0.376

*Note*: Model I: adjusted for age, gender, diabetes, hypertension, LVEF; Model II: adjusted for age, gender, diabetes, hypertension, LVEF, NTproBNP, hsCTnT, Gensini score, CRP, and d‐dimer.

Abbreviations: apo A1, apolipoprotein A1; Cr, serum creatinine; CRP, C‐reactive protein; HbA1C, glycated hemoglobin; HDL‐c, high‐density lipoprotein cholesterol; hsCTnT, high‐sensitive cardiac troponin; IL‐18, interleukin‐18; INR, international normalized ratio; LA, left atrial diameter; LDL‐c, low‐density lipoprotein cholesterol; LVDs, Left ventricular end‐diastolic dimension; LVEF, left ventricular ejection; NT‐proBNP, N‐terminal pro‐B‐type natriuretic peptide; WBC, white blood cell.

^a^ Statistically significant.

In our study, minor events like heart failure exacerbation and instances of recurrent UA (30 cases) were more prevalent than major events such as cardiovascular deaths, AMI, and stroke (11cases). To prioritize the most clinically significant aspects within this composite, we employed the WR analysis. This method highlights the most critical event, considering its clinical significance and timing.^21^, ^22^ Comparing the high and low IL‐18 groups involved logistic regression‐based propensity score adjustment, matching multiple variables.^18^ Paired patients were evaluated for specific events, with priority given to mortality, followed by AMI, stroke, heart failure readmission, and recurrent UA during the follow‐up. The results showed 24 wins (NW = 24) for the high IL‐18 group, 11 wins (NL = 11) for the low IL‐18 group, and 44 ties (N = 44). The WR for adverse clinical events in the high IL‐18 group was 2.19 (95% CI: 1.55−3.24, *Z* = 2.325, *p* = 0.010, Supporting Information S1: Table 4).

### 3.4 Association between IL‐18 and the risk of adverse clinical events

**Figure 2:**
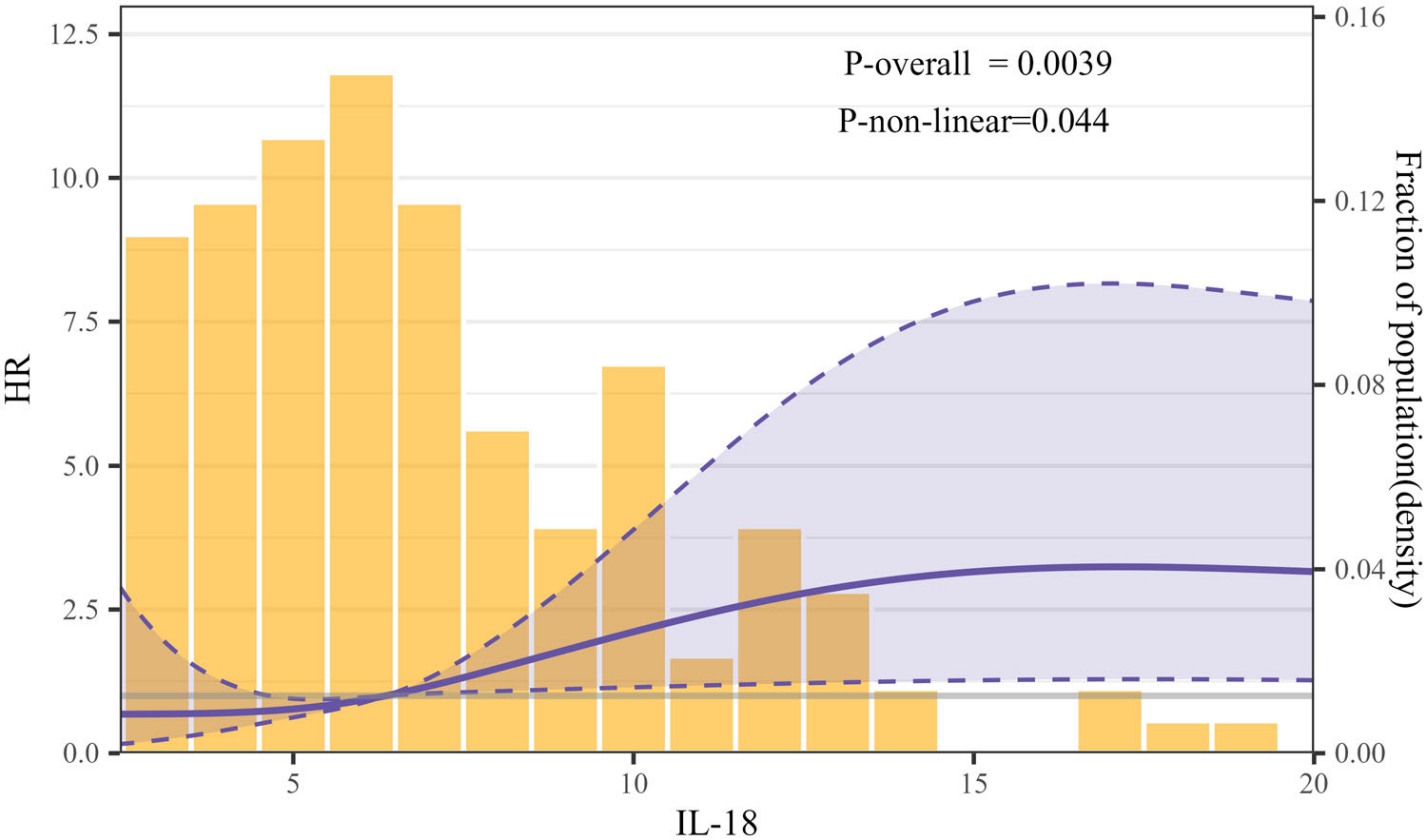
Multivariable adjusted hazard ratios (HR) for adverse clinical events according to levels of IL‐18 on a continuous scale. Solid purple lines are multivariable adjusted HR, with dashed purple lines showing 95% confidence intervals derived from restricted cubic spline regressions with four knots. The median of IL‐18 6.36 × 10^2^ pg/mL was selected as the reference level. Yellow histograms show the fraction of the population with different levels of IL‐18(10^2^ pg/mL). Adjusted for age, gender, diabetes, hypertension, LVEF, NTproBNP, hsCTnT, LVPW, Gensini scores, and CRP. CRP, C‐reactive protein; LVEF, left ventricular ejection fraction; LVPW, left ventricular posterior wall.

RCS analysis was employed to explore the relationship between IL‐18 levels and the risk of adverse clinical events. Figure 2 illustrates a nonlinear association between IL‐18 and the risk of adverse clinical events (*p*‐nonlinear = 0.044, *p*‐overall = 0.0039). Utilizing the “segmented” package, we identified the inflection point for IL‐18 at 14.7 × 10² pg/mL. On the left side of this inflection point, the risk of composite endpoints increased with rising IL‐18 levels (HR = 1.16, 95% CI: 1.05−1.29, *p* < 0.033). Conversely, on the right side of the inflection point, the fluctuation was not statistically significant (HR = 0.96, 95% CI: 0.89−1.05, *p* = 0.377, Supporting Information S1: Table 5).

**Figure 3:**
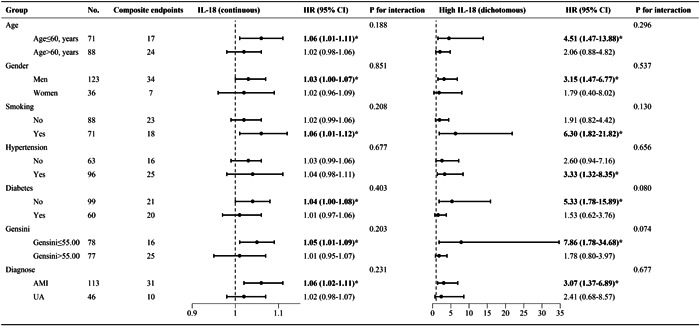
IL‐18 as a predictor of adverse clinical events occurrence in different subgroups. The HR (hazard ratio) values for each subgroup were adjusted for age, gender, hypertension, diabetes, and LVEF. *Statistically significant. AMI, acute myocardial infarction; LVEF, left ventricular ejection fraction; UA, unstable angina.

Subgroup analyses were conducted to investigate the influence of various factors on the relationship between IL‐18 and the occurrence of adverse clinical events among the follow‐up population. These factors included age, gender, history of hypertension, diabetes, smoking, median Gensini score, and the type of ACS. Additionally, we explored this association using IL‐18 as both a continuous and dichotomous variable. As depicted in Figure 3, the results revealed that the presence of these factors did not significantly alter the association between IL‐18 and the risk of adverse clinical events (*p* interaction >0.05) whether IL‐18 was analyzed as a continuous or dichotomous variable. However, more pronounced effects were observed in specific subgroups. Notably, the association was stronger in patients under the age of 60, males, individuals without diabetes or with a history of smoking, those with a Gensini score of ≤55.00, or those diagnosed with AMI (all *p* ≤ 0.05). Furthermore, elevated IL‐18 levels were linked to an increased risk of adverse clinical events among participants with a history of hypertension when IL‐18 was used as a dichotomous variable (*p* ≤ 0.05).

## 4 DISCUSSION

The objective of this study was to assess the circulating levels of IL‐1β and IL‐18 during the progression of ACS and to determine their association with subsequent clinical events in ACS patients. Our findings revealed that patients diagnosed with ACS displayed markedly higher levels of IL‐18 compared to the control group, whereas the levels of IL‐1β remained consistent. Moreover, the high IL‐18 group had a significantly greater adverse clinical events rate, marked by higher IL‐18 levels in participants who experienced composite endpoints. Significantly, we have unveiled a substantial and nonlinear association between elevated IL‐18 levels and an increased risk of adverse clinical events in ACS patients. WR analysis, emphasizing the most pivotal event, consistently confirmed these findings. Furthermore, subgroup analysis revealed that these associations remained robust and were not significantly influenced by factors such as age, gender, history of diabetes, smoking, Gensini score, or ACS type.

The persistence of elevated inflammatory markers and low‐grade inflammation, often referred to as the residual inflammatory risk, continues to be a challenge in the management of ACS. Addressing this residual inflammatory risk has the potential to improve ACS patient outcomes and diminish the collective cardiovascular disease burden.^23^, ^24^, ^25^ Prior research has established a positive correlation between increased levels of inflammatory markers like CRP, WBC count, and IL‐16, and the progression of ACS.^26^, ^27^, ^28^ Recent studies have also emphasized the significance of pyroptosis in exacerbating inflammation through the release of proinflammatory cytokines, contributing to plaque rupture and ACS development.^29^

IL‐18, a proinflammatory cytokine implicated in the process of pyroptosis, is synthesized by various immune cells, such as macrophages, dendritic cells, and epithelial cells. Its biological effects extend to T cells, natural killer cells, and neutrophils, inciting the generation of interferon‐gamma and tumor necrosis factor‐alpha.^12^, ^30^ IL‐18 plays an intricate role in inflammatory conditions, encompassing autoimmune disorders, infectious diseases, and atherosclerosis.^31^ Given its central role, the diagnostic and prognostic capabilities of IL‐18 in the context of atherosclerosis and coronary artery disease have garnered significant clinical and research interest. In our study, patients with ACS exhibited significantly elevated levels of IL‐18 when compared to the control group. This observation is consistent with findings from the Prospective Epidemiological Study of Myocardial Infarction (PRIME), revealing that baseline IL‐18 levels were notably higher in initially healthy European men who later experienced a coronary event in comparison to the control group.^32^ However, it is important to note that these results have not been replicated in the smaller population of the MONICA/KORA Augsburg Case‐Cohort Study. Although baseline IL‐18 levels were slightly higher in individuals with incident coronary heart disease compared to noncases, this difference lacked clinical significance.^13^ The observed disparity between studies is presumed to be primarily due to variations in the populations studied.

Both IL‐18 and IL‐1β play crucial roles in the pyroptosis pathway, which prompts the query of whether using IL‐1β or these cytokines collectively as a panel could serve as prognostic indicators for ACS. While Oprescu et al. reported elevated IL‐1β levels in ACS patients when compared to the control group,^33^ our findings showed relatively stable IL‐1β levels. Significantly, our study encountered null values for IL‐1β levels, leading to their exclusion in the initial analysis. Substantial sensitivity analyses, employing a commonly used replacement strategy for these null values, indicated that their removal did not result in statistically significant differences in IL‐1β between the groups. Several plausible explanations have been proposed for the null values. First, it's worth considering that the measurement of plasma IL‐1β levels may be unreliable, as indicated in previous studies.^34^ Furthermore, even in situations marked by heightened IL‐1 activity, circulating levels of IL‐1β are typically low, potentially rendering them insufficient for reliable analysis and quantification.^35^

Importantly, our study revealed that during a median follow‐up duration of 25 months, ACS patients with IL‐18 levels exceeding the median exhibited a notably increased risk of adverse cardiovascular events compared to those with IL‐18 levels below the median. This association persisted even after accounting for clinical risk factors, including d‐dimer, considered as a predictor of major clinical events in ACS patients.^36^ To prioritize cardiovascular deaths over nonfatal events, we adjusted our analyses using a WR approach for composite endpoint analysis. Despite this recalibration, individuals with high IL‐18 levels continued to demonstrate an elevated risk of adverse clinical events, affirming our initial findings. As we delved deeper into the relationship between IL‐18 levels and the risk of adverse cardiovascular events, we identified a nonlinear association. Our findings align with the prevailing understanding that the outcome of ACS is intricately linked to inflammatory processes.^3^, ^37^ Indeed, IL‐18 has shown promising potential as a prognostic biomarker in the context of ACS.^12^, ^38^ Multiple studies have emphasized circulating IL‐18 as a robust predictor of cardiovascular outcomes in ACS, consistent with our findings.^14^, ^15^, ^39^ However, the conflicting results observed in studies by Choi and Tiret suggest that the influence of IL‐18 levels cardiovascular mortality over the long term may be less significant or that their predictive value diminishes over time.^40^, ^41^ These variations are likely attributable to differences in patient cohorts and the timing of sample collection. Choi's study examined IL‐18 levels within 2 weeks following an ACS episode, whereas Tiret's research involved individuals with coronary heart disease, not exclusively ACS cases.

Subgroup analysis highlighted IL‐18's consistent link to composite endpoints across demographics and clinical factors like age, gender, medical history, and ACS type. In our study, its impact was stronger in younger males, non‐diabetics, or those with a smoking history. Notably, IL‐18's prognostic value for ACS was less pronounced in the elderly, possibly due to delayed inflammatory responses.^42^, ^43^ However, among nonsmokers, IL‐18 didn't elevate risk, suggesting a potential counteraction of its adverse effects on cardiovascular risk in this subgroup. This aligns with smoking's established impact on adverse cardiovascular outcomes.^44^, ^45^ Surprisingly, elevated IL‐18 didn't significantly correlate with adverse events in patients with diabetes, higher Gensini scores, or diagnosed with UA, likely due to smaller subgroup sizes.

## 5 LIMITATIONS

We recognize several limitations inherent in this study that warrant acknowledgment. First and foremost, the relatively small sample size may potentially limit the broader applicability and generalization of our findings to a more extensive and diverse population of patients with ACS. The results should be interpreted cautiously, keeping in mind the constraints imposed by the sample size. Moreover, despite the inclusion of a relatively extended follow‐up period, it's essential to emphasize that the number of major adverse clinical events (cardiovascular‐related fatalities, AMI, and strokes) observed during this time frame remained limited. This presented challenges in constructing an optimized multivariable regression model. Consequently, our focus shifted toward capturing extended endpoint events such as heart failure exacerbation and recurrent UA to strengthen our statistical model. The scarcity of these major events may impact the statistical power to detect subtle differences, emphasizing the need for prolonged observational periods and larger cohort studies specifically targeting critical cardiovascular events to bolster the robustness of our findings. Finally, the absence of repeated measurements of IL‐18 during the follow‐up period presents a challenge in establishing a comprehensive temporal profile of IL‐18 levels in ACS patients. Its reproducibility and stability over time remain unknown. Such longitudinal data could provide a more dynamic understanding of how IL‐18 changes over time and its potential influence on clinical outcomes. The lack of serial measurements is a limitation that future studies might consider addressing to enhance our insights into the evolving role of IL‐18 in ACS progression and prognosis.

## 6 CONCLUSIONS

In summary, our study reveals a robust link between elevated IL‐18 levels and an increased risk of adverse clinical events in ACS patients. Notably, this relationship exhibits a nonlinear pattern. Subgroup analysis reinforces the robustness of these associations and indicates that they are not significantly affected by factors such as age, gender, history of diabetes, smoking, Gensini score, or ACS type. Additional research is warranted to thoroughly clarify the role of IL‐18 in ACS and its differential impact across various patient subgroups.

## AUTHOR CONTRIBUTIONS

Chenchun Xiong and Qiaoting Yu were responsible for the initial draft of the manuscript. Song Liu and Qiaoting Yu contributed to data collection. Chenchun Xiong, Qiaoting Yu, and Jianhui Zhang analyzed the data. Tianyi Ma and Feng Gao were responsible for conducting the coronary angiography. Chenchun Xiong and Suifeng Liu conducted the statistical analysis. Suifeng Liu provided essential manuscript revisions. All authors reviewed and approved the final version.

## CONFLICT OF INTEREST STATEMENT

The authors declare no conflict of interest.

## Supporting information

Supporting information.

## Data Availability

Upon a reasonable inquiry, the corresponding author is ready to supply all the data employed in this study.
