# Supplementary material for: Prognostic significance of IL‐18 in acute coronary syndrome patients
Source: Clin Cardiol. 2024 Jan 29;47(2):e24229. doi: 10.1002/clc.24229 (PMC10823553; doi:10.1002/clc.24229)
Supplement: Supplementary file 1 — Supporting information. [file CLC-47-e24229-s001.docx]

**Supplemental Table 1.** Best-worst case analysis to hande measurements of IL-1β below the detection limit

|  | IL-1β, pg/mL |  |  |
| --- | --- | --- | --- |
| Replacement value | Control (n=159) | ACS (n=159) | P |
| No replacement^#^ | 0.12 [0.06, 0.30] | 0.15 [0.05, 0.48] | 0.726 |
| LOD | 0.03 [0.03, 0.06] | 0.03 [0.03, 0.14] | 0.096 |
| LOD/2 | 0.02 [0.02, 0.08] | 0.02 [0.02, 0.14] | 0.053 |

LOD, the limit of detection; IL-1β, interleukin-1β. ^#^Not detectable (0.033 pg/mL) in 191 (108 in the control group and 83 in the ACS group) out of 318 participants, excluded from the analyses.

**Supplemental Table 2.** Clinical characteristics of the study population across IL-18

| Characteristics | Overall | IL-18 ≤Median | IL-18 >Median | P |
| --- | --- | --- | --- | --- |
| No. of participants | 159 | 80 | 79 |  |
| Events, n (%) | 41 (25.8) | 12 (15.0) | 29 (36.7) | 0.003* |
| **Sociological characteristics** |  |  |  |  |
| Age, years | 62.98 (12.43) | 62.16 (13.17) | 63.81 (11.66) | 0.405 |
| Men, n (%) | 123 (77.4) | 60 (75.0) | 63 (79.7) | 0.599 |
| Smoking, n (%) | 71 (44.7) | 35 (43.8) | 36 (45.6) | 0.943 |
| **Comorbidities** |  |  |  |  |
| Hypertension, n (%) | 96 (60.4) | 45 (56.2) | 51 (64.6) | 0.364 |
| Diabetes, n (%) | 60 (37.7) | 28 (35.0) | 32 (40.5) | 0.581 |
| kidney disease, n (%) | 31 (19.5) | 12 (15.0) | 19 (24.1) | 0.215 |
| **Echocardiography** |  |  |  |  |
| LA, mm | 39.00 [36.00, 41.00] | 38.00 [36.00, 40.25] | 39.00 [36.00, 41.00] | 0.453 |
| LVDs, mm | 32.00 [29.00, 34.50] | 32.00 [28.00, 34.25] | 33.00 [29.00, 34.50] | 0.372 |
| LVEF, % | 61.00 [52.50, 66.00] | 62.00 [53.75, 66.00] | 59.00 [51.50, 64.00] | 0.136 |
| **Laboratory values** |  |  |  |  |
| Cardiac parameters |  |  |  |  |
| hsCTnT, pg/mL | 490.10 [101.45, 2680.00] | 403.00 [52.93, 2520.25] | 870.00 [121.60, 3129.50] | 0.333 |
| NT-proBNP, pg/mL | 331.00 [78.25, 1140.50] | 297.60 [73.20, 1056.10] | 367.00 [89.05, 1236.00] | 0.539 |
| Inflammatory markers |  |  |  |  |
| IL-18, 10^2^pg/mL | 6.36 [4.46, 9.71] | 4.46 [3.09, 5.30] | 9.88 [7.51, 12.32] | <0.001* |
| CRP, mg/L | 4.08 [1.94, 18.22] | 4.08 [1.68, 19.72] | 4.08 [2.02, 17.14] | 0.567 |
| WBC, 10^9^/L | 8.67 [7.00, 11.32] | 8.41 [6.91, 11.13] | 9.55 [7.76, 11.46] | 0.121 |
| Coagulation parameters |  |  |  |  |
| INR | 1.03 [0.97, 1.14] | 1.03 [0.97, 1.18] | 1.03 [0.96, 1.10] | 0.472 |
| Fibrinogen, g/L | 3.24 [2.78, 4.04] | 3.37 [2.89, 4.10] | 3.18 [2.66, 3.92] | 0.172 |
| D-dimer, mg/L | 0.34 [0.20, 0.62] | 0.31 [0.19, 0.48] | 0.37 [0.23, 0.75] | 0.028* |
| Lipid profile |  |  |  |  |
| HDL-c, mmol/L | 1.08 [0.92, 1.27] | 1.08 [0.92, 1.20] | 1.06 [0.92, 1.27] | 0.940 |
| LDL-c, mmol/L | 3.20 [2.63, 3.99] | 3.20 [2.63, 3.93] | 3.20 [2.65, 4.00] | 0.996 |
| apoA1, g/L | 1.14 (0.25) | 1.15 (0.23) | 1.13 (0.28) | 0.555 |
| Other parameters |  |  |  |  |
| Cr, µmol/L | 79.90 [65.80, 94.15] | 79.25 [65.38, 90.82] | 80.20 [68.20, 100.15] | 0.531 |
| HbA1C, % | 6.30 [5.80, 7.30] | 6.40 [5.77, 7.20] | 6.30 [5.80, 7.70] | 0.767 |
| **Gensini** | 55.00 [32.75, 82.00] | 58.00 [37.00, 86.00] | 50.00 [32.00, 80.00] | 0.303 |

Data are presented as median [IQR], mean (average) or number (%), as appropriate. Events including cardiovascular death, acute myocardial infarction, stroke, heart failure readmission and recurrent angina. LA, left atrial diameter; LVDs, Left ventricular end-diastolic dimension; LVEF, left ventricular ejection; IL-18, interleukin-18; WBC, white blood cell; CRP, C-reactive protein; INR, international normalized ratio; HDL-c: LDL-c: low-density lipoprotein cholesterol; high-density lipoprotein cholesterol; apo A1, apolipoprotein A1; Cr, serum creatinine; HbA1C, glycated hemoglobin. *: statistically significant.

**Supplemental Table 3.** Clinical characteristics of the study population across adverse clinicl events occurrence

| Characteristics | Overall | no Event | Event | P |
| --- | --- | --- | --- | --- |
| No. of participants | 159 | 118 | 41 |  |
| **Sociological characteristics** |  |  |  |  |
| Age, years | 64.00 [54.50, 72.00] | 63.00 [53.00, 72.00] | 66.00 [55.00, 77.00] | 0.157 |
| Men, n (%) | 123 (77.4) | 89 (75.4) | 34 (82.9) | 0.440 |
| Smoking, n (%) | 71 (44.7) | 53 (44.9) | 18 (43.9) | 1.000 |
| **Comorbidities** |  |  |  |  |
| Hypertension, n (%) | 96 (60.4) | 71 (60.2) | 25 (61.0) | 1.000 |
| Diabetes, n (%) | 60 (37.7) | 40 (33.9) | 20 (48.8) | 0.132 |
| kidney disease, n(%) | 31 (19.5) | 19 (16.1) | 12 (29.3) | 0.109 |
| **Echocardiography** |  |  |  |  |
| LA, mm | 39.00 [36.00, 41.00] | 38.50 [36.00, 40.00] | 39.00 [36.00, 43.00] | 0.138 |
| LVDs, mm | 32.00 [29.00, 34.50] | 32.00 [29.00, 34.00] | 33.00 [28.00, 38.00] | 0.443 |
| LVEF, % | 61.00 [52.50, 66.00] | 62.00 [54.25, 66.00] | 58.00 [41.00, 64.00] | 0.013* |
| **Laboratory values** |  |  |  |  |
| Cardiac function parameters |  |  |  |  |
| hsCTnT, pg/mL | 490.10 [101.45, 2680.00] | 480.05 [47.42, 2631.25] | 501.00 [146.00, 3131.00] | 0.529 |
| NT-proBNP, pg/mL | 331.00 [78.25, 1140.50] | 301.10 [70.95, 1054.82] | 537.00 [94.40, 1692.00] | 0.106 |
| Inflammatory markers |  |  |  |  |
| IL-18, 10^2^pg/mL | 6.36 [4.46, 9.71] | 5.96 [3.97, 8.10] | 8.53 [5.98, 11.53] | 0.002* |
| CRP, mg/L | 4.08 [1.94, 18.22] | 4.08 [1.77, 13.41] | 5.54 [2.62, 25.58] | 0.106 |
| WBC, 10^9^/L | 8.67 [7.00, 11.32] | 8.59 [6.92, 11.20] | 9.16 [8.12, 11.49] | 0.203 |
| Coagulation parameters |  |  |  |  |
| INR | 1.03 [0.97, 1.14] | 1.04 [0.97, 1.17] | 1.01 [0.96, 1.07] | 0.336 |
| Fibrinogen, g/L | 3.24 [2.78, 4.04] | 3.24 [2.81, 3.98] | 3.41 [2.76, 4.22] | 0.958 |
| D-dimer, mg/L | 0.34 [0.20, 0.62] | 0.32 [0.19, 0.54] | 0.41 [0.25, 0.91] | 0.021 |
| Lipid profile |  |  |  |  |
| HDL-c, mmol/L | 1.08 [0.92, 1.27] | 1.09 [0.92, 1.27] | 1.04 [0.85, 1.21] | 0.380 |
| LDL-c, mmol/L | 3.20 [2.63, 3.99] | 3.28 [2.65, 3.99] | 3.05 [2.58, 3.72] | 0.382 |
| apoA1, g/L | 1.14 (0.25) | 1.16 (0.25) | 1.08 (0.26) | 0.058 |
| Other parameters |  |  |  |  |
| Cr, µmol/L | 79.90 [65.80, 94.15] | 78.75 [65.32, 91.80] | 83.10 [70.60, 106.10] | 0.150 |
| HbA1C, % | 6.30 [5.80, 7.30] | 6.20 [5.80, 7.20] | 6.60 [6.00, 7.70] | 0.135 |
| **Gensini** | 55.00 [32.75, 82.00] | 50.00 [28.00, 80.75] | 64.00 [47.00, 86.00] | 0.024* |

Data are presented as median [IQR], mean (average) or number (%), as appropriate. MACE, major adverse cardiovascular events (worsening heart failure, cardiovascular death, stroke, recurrent angina and acute myocardial infarction). LA, left atrial diameter; LVDs, Left ventricular end-diastolic dimension; LVEF, left ventricular ejection; IL-18, interleukin-18; CRP, C-reactive protein; WBC, white blood cell; INR, international normalized ratio; HDL-c: LDL-c: low-density lipoprotein cholesterol; high-density lipoprotein cholesterol; apo A1, apolipoprotein A1; Cr, serum creatinine; HbA1C, glycated hemoglobin. *: statistically significant.

**Supplemental Table 4.** Results using a matched pairs approach to the win ratio

|  | High IL-18 | Low IL-18 |  |
| --- | --- | --- | --- |
| Wins/Losses | Wins | Losses | WR (95% CI) |
| Cardiovascular death | 3 | 2 |  |
| AMI | 2 | 1 |  |
| Stroke | 2 | 4 |  |
| Heart failure hospitalization | 8 | 1 |  |
| Recurrent UA | 9 | 3 |  |
| **Composite outcome** | **24** | **11** | **2.19 (1.55-3.24) *** |

IL-18, interleukin-18; WR, win ratio; AMI, acute myocardial infarction; UA, unstable angina. Hierarchy: Cardiovascular death > Myocardial infarction > Stroke > Heart failure hospitalization > Recurrent angina pectoris. *: statistically significant.

**Supplemental Table 5.** Analyzing the relationship between IL-18 and adverse clinical events using 2 piece-wise linear regression models

| IL-18（10^2^pg/mL） | IL-18 | |
| --- | --- | --- |
|  | HR (95% CI) | P |
| IL-18<14.7 | 1.16 (1.05-1.29) | 0.005* |
| IL-18≥14.7 | 0.96 (0.89-1.05) | 0.377 |

IL-18, interleukin-18; HR, Hazard ratio; CI, confidence interval.
